# Heterogeneous antiretroviral drug distribution and HIV/SHIV detection in the gut of three species

**DOI:** 10.1126/scitranslmed.aap8758

**Published:** 2019-07-03

**Authors:** Corbin G. Thompson, Elias P. Rosen, Heather M. A. Prince, Nicole White, Craig Sykes, Gabriela de la Cruz, Michelle Mathews, Claire Deleage, Jacob D. Estes, Paige Charlins, Leila R. Mulder, Martina Kovarova, Lourdes Adamson, Shifali Arora, Evan S. Dellon, Anne F. Peery, Nicholas J. Shaheen, Cynthia Gay, David C. Muddiman, Ramesh Akkina, J. Victor Garcia, Paul Luciw, Angela D. M. Kashuba

**Affiliations:** 1 Division of Pharmacotherapy and Experimental Therapeutics, University of North Carolina at Chapel Hill, Chapel Hill, NC, USA.; 2 School of Medicine, University of North Carolina at Chapel Hill, Chapel Hill, NC, USA.; 3 Division of Infectious Diseases, Center for AIDS Research, University of North Carolina at Chapel Hill, Chapel Hill, NC, USA.; 4 AIDS and Cancer Virus Program, Frederick National Laboratory for Cancer Research, Leidos Biomedical Research Inc., Frederick, MD, USA.; 5 Vaccine and Gene Therapy Institute, Oregon Health & Science University, Beaverton, OR, USA.; 6 Department of Microbiology, Immunology and Pathology, Colorado State University, Fort Collins, CO, USA.; 7 Department of Pathology, Microbiology and Immunology, School of Veterinary Medicine, University of California, Davis, Davis, CA, USA.; 8 W.M. Keck FTMS Laboratory for Human Health Research, Department of Chemistry, North Carolina State University, Raleigh, NC, USA.

## Abstract

HIV replication within tissues may increase in response to a reduced exposure to antiretroviral drugs. Traditional approaches to measuring drug concentrations in tissues are unable to characterize a heterogeneous drug distribution. Here, we used mass spectrometry imaging (MSI) to visualize the distribution of six HIV antiretroviral drugs in gut tissue sections from three species (two strains of humanized mice, macaques, and humans). We measured drug concentrations in proximity to CD3^+^ T cells that are targeted by HIV, as well as expression of HIV or SHIV RNA and expression of the MDR1 drug efflux transporter in gut tissue from HIV-infected humanized mice, SHIV-infected macaques, and HIV-infected humans treated with combination antiretroviral drug therapy. Serial 10-μm sections of snap-frozen ileal and rectal tissue were analyzed by MSI for CD3^+^ T cells and MDR1 efflux transporter expression by immunofluorescence and immunohistochemistry, respectively. The tissue slices were analyzed for HIV/SHIV RNA expression by in situ hybridization and for antiretroviral drug concentrations by liquid chromatography–mass spectrometry. The gastrointestinal tissue distribution of the six drugs was heterogeneous. Fifty percent to 60% of CD3^+^ T cells did not colocalize with detectable drug concentrations in the gut tissue. In all three species, up to 90% of HIV/SHIV RNA was found to be expressed in gut tissue with no exposure to drug. These data suggest that there may be gut regions with little to no exposure to antiretroviral drugs, which may result in low-level HIV replication contributing to HIV persistence.

## INTRODUCTION

The persistence of HIV despite treatment with combination antiretroviral therapy is the major obstacle to eradication of this disease, necessitating lifelong therapy in HIV-infected individuals. Rebound plasma viremia after treatment cessation is thought to be secondary to reactivation of latently infected memory T cell populations (1–4). However, it has been hypothesized that residual HIV replication from productively infected T cells within tissue reservoirs may also contribute to viral rebound upon treatment interruption (5).

The evidence for continued production of replication-competent virions within certain anatomical sites has been extensively reviewed (6, 7). Several tissue compartments, such as the central nervous system (8), male and female genital tracts (9, 10), peripheral lymph nodes (11), and gut-associated lymphoid tissue (GALT) (10), have been implicated as potential tissue reservoirs and have recently been characterized in nonhuman primate (NHP) models (12). The consequences of persistent active HIV replication in GALT, such as prolonged immune dysregulation and incomplete CD4^+^ T cell reconstitution during combination antiretroviral therapy, are different from the consequences in other anatomical compartments (13). The mechanisms underlying HIV persistence in tissue reservoirs like GALT are unclear, but suboptimal antiretroviral drug penetration into tissues has been suggested as one potential cause (14). Inadequate tissue exposure to antiretroviral drugs may explain the persistence of HIV replication.

Traditional methods for measuring the penetration of small molecules into tissues are limited in their capacity to measure heterogeneity of drug distribution within tissues. Liquid chromatography–mass spectrometry (LC-MS) analysis of tissue homogenates has been the gold standard for antiretroviral drug tissue analysis but provides only an aggregate concentration of drug over the entire tissue. If HIV replication occurs focally, for example, in a single lymphoid follicle, LC-MS analysis may not accurately represent antiretroviral drug concentrations at the site of action. Some groups have isolated mononuclear cell populations from tissues before performing LC-MS (14). Although this method is an improvement over analysis of tissue homogenates, it does not fully account for drug lost during mononuclear cell isolation (15) and cannot distinguish between mononuclear cell populations from different sites within the tissue.

Mass spectrometry imaging (MSI) can be used to quantitate small molecules at discrete tissue sample locations while maintaining spatial information across the entirety of a sectioned tissue sample, which allows the analyte distribution within a sample to be visualized (16, 17). An advantage of MSI is the ability of this method to spatially correlate antiretroviral drug distribution with sites of potential HIV replication in serial tissue sections. Focal HIV RNA expression in tissue areas with low antiretroviral drug concentrations could indicate areas where there is active viral replication.

In addition to advancing our understanding of how antiretroviral drugs become distributed in tissues, MSI can provide information about whether antiretroviral drug distribution in tissues differs among preclinical animal models and between animals and humans. Although the development of any new therapeutic agent requires testing in animal models, efficacy end points may differ if the within-tissue drug distribution is different between the animal models and humans.

Here, we used MSI to visualize the distribution of antiretroviral drugs from several therapeutic classes in gastrointestinal tissues from HIV-infected humanized mice, SHIV (simian/human immunodeficiency virus)–infected macaques, and HIV-infected human subjects. We assessed the colocalization of antiretroviral drugs with HIV target cells and viral RNA expression. Then, we determined the extent to which antiretroviral drug distribution was associated with potential sites of viral replication. We also evaluated antiretroviral drug distribution in the context of drug efflux transporter expression by gut epithelial cells.

## RESULTS

### Demographics of patients in clinical study

Between April 2016 and August 2017, a total of five HIV-infected women taking some combination of tenofovir disoproxil fumarate/emtricitabine, efavirenz, atazanavir, raltegravir, and maraviroc completed the clinical study (NCT02641444; summary demographics are shown in table S1). Participants had a median age of 53 years and a median body mass index (BMI) of 39.1; four of five women were African-American. Menopause status was evenly distributed with two women premenopause and three women postmenopause. All five participants had been HIV positive for >5 years (median, 18 years) and had been on their current combination antiretroviral drug therapy regimen for a median of 8 years. Participants had well-controlled HIV infection as evidenced by undetectable (<50 copies/ml) plasma viremia and high CD4 T cell counts (median, 753 cells/mm^3^). A single adverse event (grade 1 headache) was observed.

### Antiretroviral drug penetration in ileal and rectal tissue

Plasma and tissue combination antiretroviral therapy concentrations measured by LC-MS in this study and our previous study (18) were used to generate tissue penetration ratios for each drug in ileum and rectum biopsies obtained by colonoscopy from five HIV-infected participants (table S2). In human ileum tissue, penetration of tenofovir was >10 times higher compared to ileum tissue in mice or NHPs (*P* = 0.007; table S2) (18). Penetration of tenofovir, efavirenz, atazanavir, raltegravir, and maraviroc into human ileum was two- to sevenfold higher compared to the ileum of mice (*P* < 0.05) and was similar to penetration of the ileum of NHPs (table S2) (18). Penetration of tenofovir into the human rectum was >10 times greater compared to the rectum of mice (*P* = 0.003). Penetration of efavirenz, atazanavir, raltegravir, and maraviroc was 2- to 10-fold higher in human rectum compared to mouse rectum (*P* < 0.05) and was similar to the penetration into the rectum of NHPs (table S2) (18).

### Heterogeneous antiretroviral drug distribution in gut tissue from three species

**Fig. 1.**
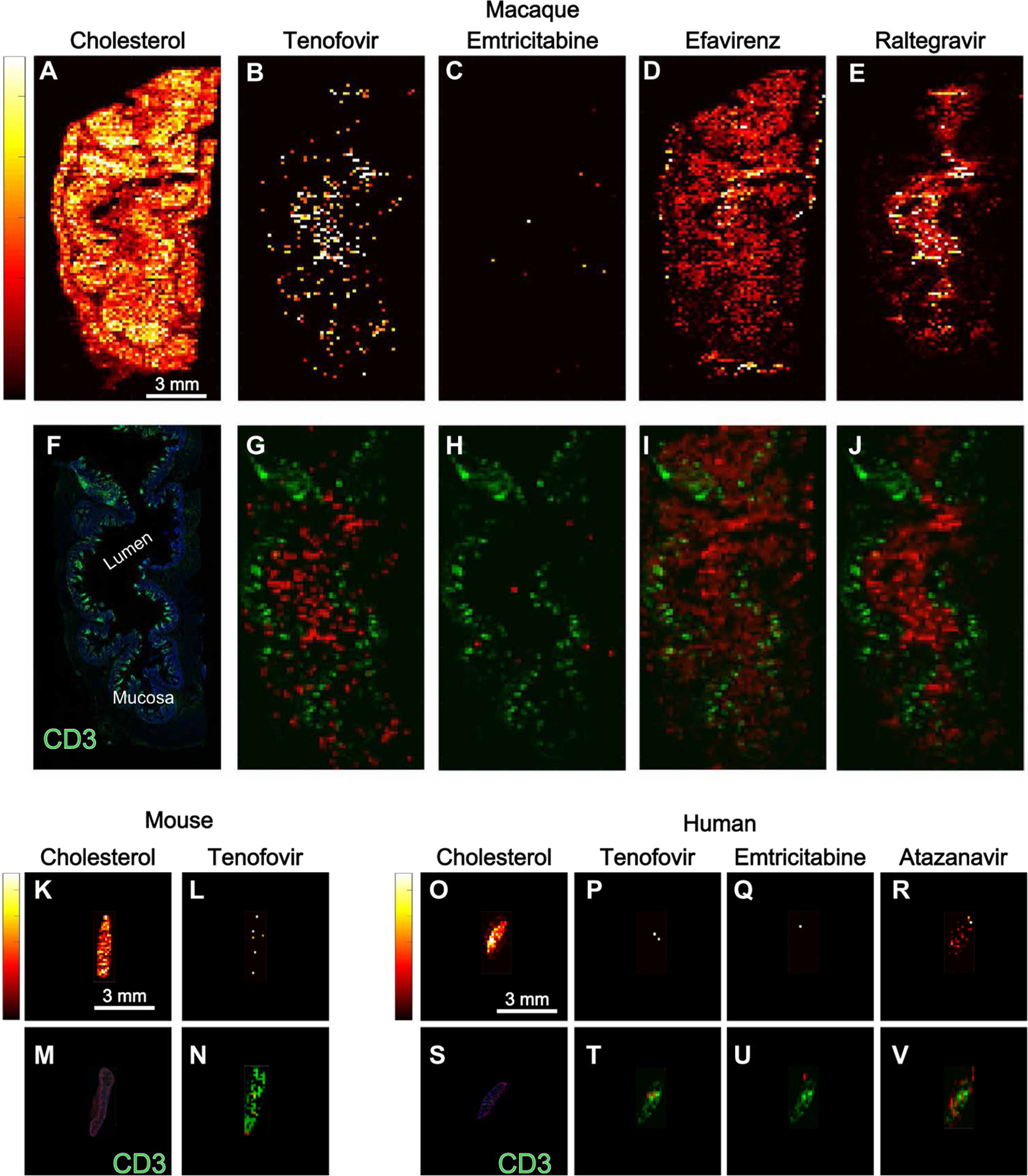
Antiretroviral drug distribution in gut tissue from three species. Representative images of antiretroviral drug distribution in a single cross-sectional 10-μm slice of ileum from an uninfected macaque, an HIV-infected humanized BLT mouse, and an HIV-infected human measured using MSI are shown. A total of 24 macaques, 98 mice, and 10 human tissue slices were imaged by MSI. (**A** to **J**) Macaque. (**K** to **N**) Mouse. (**O** to **V**) Human. Relative drug abundance is indicated by the color scale: Lighter colors (yellow and white) are associated with higher drug concentrations than darker colors (red and black). Endogenous cholesterol distribution is shown in macaque ileum in (A) to demonstrate tissue orientation for the remaining images. Drug distribution images for all antiretroviral drugs administered to this macaque (B to E) are overlaid with the localization of CD3^+^ T cells from an adjacent tissue section (F), and the colocalization is shown in (G) to (J) (drug, red; CD3^+^ T cells, green). Similar images of antiretroviral drug (tenofovir) concentration on its own or overlaid with CD3^+^ T cell localization are shown for an HIV-infected BLT mouse (K to N) and an HIV-infected human (O to V). Scale bars to the left of each endogenous cholesterol image apply to all antiretroviral drug distribution images for that tissue slice. In the BLT mouse ileum slice (K and L), tenofovir was the only drug detected by MSI and was overlaid with the CD3^+^ T cell image (N). In the human ileum tissue section (O to R), all three drugs contained in the patient’s antiretroviral drug regimen were detected by MSI. (T) to (V) show these images overlaid with CD3^+^ T cell localization (S).

Heterogeneous distribution of antiretroviral drugs was observed in 10-μm gut tissue sections from two strains of humanized mice, 12 macaques (6 uninfected and 6 SHIV-infected) and 5 HIV-infected humans. Representative images of antiretroviral drug distribution in the three species are shown in Fig. 1. Images of endogenous cholesterol were included to delineate tissue orientation (Fig. 1A). In uninfected macaques, tenofovir (Fig. 1B) and emtricitabine (Fig. 1C) showed disparate drug distribution patterns, with tenofovir having the highest signal in the lumen of the ileum, and low but consistent penetration into the mucosa and outer muscle layer of the ileum. In contrast, emtricitabine concentrations were below the detection limit of our MSI technique. Consistent with our previously published data (19), the distribution of efavirenz was heterogeneous throughout the ileum and rectum of uninfected macaques and was localized primarily to the mucosal layer (Fig. 1D). Conversely, raltegravir was localized to the lumen of the ileum and rectum of uninfected macaques with less penetration into the mucosal layer (Fig. 1E). We next investigated the localization of CD3^+^ T cells in the ileum and rectum of uninfected macaques (Fig. 1, G to J). Fourteen percent to 29% of all cells in the macaque ileum were calculated to be CD3^+^ T cells (Fig. 1F). Differences between the distribution of antiretroviral drugs and the localization of CD3^+^ T cells were observed in the uninfected macaque ileum and rectum.

Representative images of the gut tissue distribution of tenofovir in an HIV-infected humanized mouse are shown in Fig. 1 (K to N). Compared to uninfected macaque gut tissues, antiretroviral drug distribution was more heterogeneous in the gut tissue of two humanized mouse strains: the hu-HSC-Rag (*n* = 36) and bone marrow–liver–thymus (BLT) (*n* = 13) humanized mouse models. Raltegravir and emtricitabine could not be detected by infrared matrix-assisted laser desorption electrospray ionization (IR-MALDESI) in any gut tissue slices from either strain of humanized mouse, despite detection of drug concentrations in mouse plasma and tissue by LC-MS. We observed fewer detectable human CD3^+^ T cells in the HIV-infected humanized mouse gut tissue samples (1 to 3% of cells were CD3^+^ T cells; Fig. 1M) due to the limited reconstitution of human T cells into this compartment, particularly in the case of the hu-HSC-Rag mice (20). Figure 1N shows the overlay of tenofovir with CD3^+^ T cells, revealing that the low tenofovir signal had limited colocalization with the signal for CD3^+^ T cells.

Representative images for antiretroviral drug distribution in gut biopsies from HIV-infected individuals are shown in Fig. 1 (O to V). There were detectable concentrations of tenofovir and emtricitabine in the plasma (tenofovir, 36 to 236 ng/ml; emtricitabine, 63 to 612 ng/ml) and gut tissue biopsies (tenofovir, 1596 to 17,820 ng/g; emtricitabine, 146 to 2762 ng/g) measured by LC-MS. However, concentrations of these two drugs could only be detected sporadically in the gut biopsy samples by MSI, even before correcting for potential blood contamination using heme. In contrast, efavirenz, raltegravir, atazanavir, and maraviroc were detected in all human ileal and rectal biopsy samples with a broad distribution throughout the mucosa and submucosa.

**Fig. 2.**
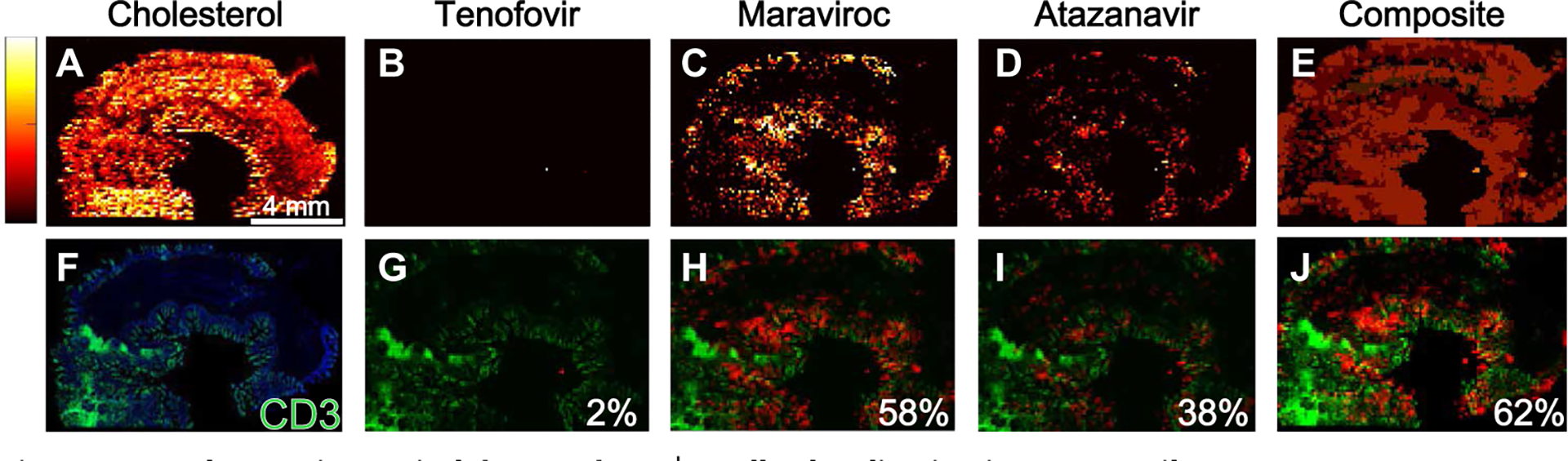
Incomplete antiretroviral drug and CD3^+^ T cell colocalization in macaque ileum. Representative images of antiretroviral drug distribution in a single cross-sectional 10-μm slice of ileum from an SHIV-infected macaque measured by MSI are shown. A total of 12 macaque ileal tissue slices were imaged by MSI. (**B** to **D**) Lighter colors (yellow and white) are associated with higher antiretroviral drug concentrations than darker colors (red and black). Endogenous cholesterol distribution is shown in (**A**) to demonstrate tissue orientation for the remaining images. (**E**) Composite image of the distribution of all three drugs. (**G** to **J**) These images were overlaid with the CD3^+^ T cell distribution from an adjacent ileal tissue slice (**F**) (drug, red; CD3^+^ T cells, green). The percentage of total CD3^+^ T cells colocalizing with tenofovir (G), maraviroc (H), or atazanavir (I) is shown at the bottom right of the respective panels. The percentage of total CD3^+^ T cells colocalizing with at least one drug is shown in (J).

Given that these drugs are given in combination for maximal antiviral efficacy, we combined discrete maps of antiretroviral drug distribution to visualize overall drug concentrations in macaque gut tissue and then inspected the overlap with the localization of CD3^+^ T cells (Fig. 2). Figure 2 shows representative MSI images assessing the extent to which antiretroviral drug combinations colocalized with CD3^+^ T cell expression measured in adjacent tissue sections of a single SHIV-infected macaque ileum. MSI images for each antiretroviral drug in the same tissue slice are shown in Fig. 2 (A to D), with a composite image representing total drug distribution in Fig. 2E. The proportion of CD3^+^ T cells that colocalized with each drug is shown in Fig. 2 (G to I), and the proportion of CD3^+^ T cells colocalizing with any of the antiretroviral drugs is shown in Fig. 2J. In this tissue slice, about 60% of CD3^+^ T cells colocalized with the distribution of either maraviroc or atazanavir, leaving 40% of the cell population unexposed to drug or exposed to drug concentrations below the limit of detection (LOD) (estimated MSI LODs: 708 fg per voxel for maraviroc and 1428 fg per voxel for atazanavir). The proportion of CD3^+^ T cells colocalized with each antiretroviral drug in ileal and rectal biopsies from humanized mice, macaques, and humans is shown in table S3. In both the ileum and rectum, tenofovir and emtricitabine distribution overlapped the least with CD3^+^ T cell localization compared to the other antiretroviral drugs, but the variability was large for all species (0 to 80% coverage). These data were confounded by the lower sensitivity of MSI regarding detection of tenofovir and emtricitabine (estimated MSI LOD: 3372 fg per voxel for tenofovir and 5026 fg per voxel for emtricitabine) and did not account for intracellular phosphorylation of these drugs to their active metabolites (tenofovir diphosphate and emtricitabine triphosphate).

Table 1 summarizes the proportion of CD3^+^ T cells that were not exposed to any antiretroviral drug for the two humanized mouse strains, macaques, and humans. No differences were observed between infected and uninfected mice and macaques, and so, these data were combined for analysis. In both the ileum and rectum, macaque and human showed the broadest CD3^+^ T cell coverage with median proportions of T cells colocalized to an antiretroviral drug of 0.5 to 0.6 (Table 1). CD3^+^ T cells in the ileum and rectum of hu-HSC-Rag mice, which represented 80% of all T cells, colocalized with maraviroc and atazanavir but not the other drugs (table S2). Human, macaque, and BLT humanized mice all had a higher proportion of CD3^+^ T cells colocalizing with each antiretroviral drug in the ileum and rectum than did hu-HSC-Rag mice (*P* < 0.01). No differences were seen in the distribution of CD3^+^ T cells between the ileum and rectum for any species (*P* > 0.05).

### Correlation of viral RNA expression with antiretroviral drug distribution

**Fig. 3.**
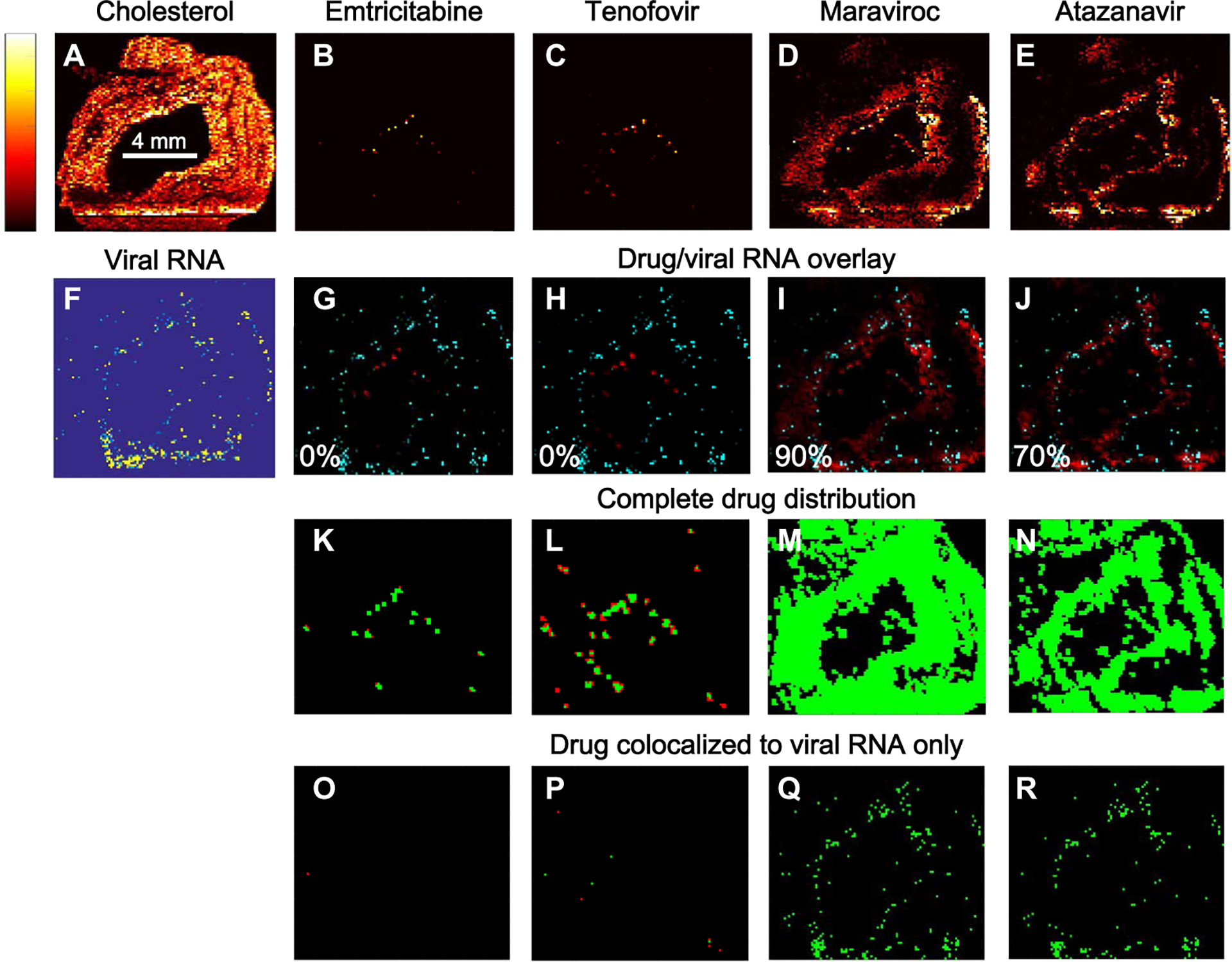
Antiretroviral drug concentrations and SHIV RNA expression. (**B** to **E**) Representative images of the distribution of four antiretroviral drugs in a single cross-sectional 10-μm slice of ileum from an SHIV-infected macaque measured by MSI. A total of 24 macaque tissue slices were imaged by MSI. Lighter colors (yellow and white) are associated with higher drug concentrations compared to darker colors (red and black). Endogenous cholesterol distribution is shown in (**A**) to enable tissue orientation for the remaining images. Images in (B) to (E) were overlaid (**G** to **J**) with a viral RNA image at matched spatial resolution from an adjacent slice (**F**) (drug, red; viral RNA, cyan). The percentage of total viral RNA colocalizing with drug is shown in the bottom left corner of each image. (**K** to **N**) Images show the distribution of each antiretroviral drug as a function of the drug’s IC_90_ value (concentrations above the IC_90_, green; concentrations below the IC_90_, red). (**O** to **R**) Similar analysis for drug that colocalizes with viral RNA.

Next, we determined whether the incomplete overlap of CD3^+^ T cells with antiretroviral drug distribution in ileum and rectum was associated with HIV or SHIV RNA expression in these tissues. MSI images of CD3^+^ T cell and drug distribution were co-registered with viral RNA images obtained from in situ hybridization (ISH) at matching image resolution. HIV or SHIV RNA expression was localized into discrete clusters within the submucosa of the ileum and rectum of all three species (fig. S1A, inset). Figure 3 shows representative MSI overlay images of antiretroviral drug distribution and viral RNA expression in SHIV-infected macaque ileum. The MSI images showed areas of viral RNA expression (Fig. 3, G to J) that did not overlap with drug distribution (Fig. 3, B to E). However, this effect was not seen consistently with all antiretroviral drugs. Emtricitabine and tenofovir were found primarily where no viral RNA was located (Fig. 3, G and H), whereas maraviroc and atazanavir were found where a large proportion of viral RNA was expressed (Fig. 3, I and J; proportion of viral RNA colocalized to drug, 0.9 and 0.7, respectively).

Penetration of antiretroviral drugs into the macaque ileum as a function of the in vitro IC_90_ (90% maximal inhibitory concentration) values for each drug is shown in Fig. 3 (K to N). Emtricitabine and tenofovir were present at concentrations below their respective IC_90_ values (Fig. 3, K and L, respectively, red); very few tissue regions had antiretroviral drugs above this threshold (green). Given that the MSI LODs for emtricitabine and tenofovir were lower than the IC_90_ values, all areas of a tissue section where these drugs were not measured were assumed to have concentrations below the IC_90_. Conversely, maraviroc, efavirenz, and atazanavir concentrations were primarily above their IC_90_ values. The distribution of individual drugs localized to regions of viral RNA expression is shown in Fig. 3 (O to R). Many of the tissue regions where emtricitabine and tenofovir were colocalized with viral RNA expression were at concentrations below the IC_90_ values for these drugs; maraviroc and atazanavir concentrations remained above this threshold in most cases. There were, however, several tissue regions where maraviroc and viral RNA expression was colocalized at sub-IC_90_ concentrations of drug (Fig. 3Q).

Table 2 shows the extent of antiretroviral drug distribution compared to viral RNA expression for HIV-infected humanized mice, SHIV-infected macaques, and HIV-infected human patients. Unlike our results for CD3^+^ T cell distribution, the localization of viral RNA expression with drug distribution was high (median values, 10 to 100%), with the ileum and rectum of some mice demonstrating complete overlap for one or more antiretroviral drugs (after correction for potential blood contamination based on the heme b signal; Table 2). However, observed variability was also high, with overlap ranging from 0 to 100%, depending on the species (Table 2). For hu-HSC-Rag humanized mice, viral RNA expression and drug distribution overlap were lower than for BLT humanized mice and macaques (0% versus 50 to 90%; *P* < 0.01; Table 2). Results in the ileum and rectum were similar for all species.

Table 3 summarizes the calculated concentrations for each antiretroviral drug closest to the region of peak viral RNA expression measured by MSI. These concentrations surpassed in vitro IC_90_ values for most of the drugs except for tenofovir and emtricitabine, which were not detected in many samples. The lack of detection of emtricitabine and tenofovir, which was consistent across species and between ileum and rectum, may reflect the limitations of MSI because these drugs achieved high tissue concentrations when measured with LC-MS (21, 22). Tenofovir and emtricitabine concentrations in serial tissue slices were more frequently detected by LC-MS than by MSI but were still below their respective IC_90_ values. Tenofovir diphosphate and emtricitabine triphosphate measured by LC-MS showed different detection rates in ileum and rectum, with tenofovir diphosphate being readily detected and emtricitabine triphosphate being infrequently detected in the three species. Efavirenz and raltegravir were only sporadically detected in the humanized mice (efavirenz, 12%; raltegravir, 35%) despite being readily detectable in macaques and humans (100%) at concentrations that greatly exceeded reported IC_90_ values. Maraviroc and atazanavir were detected with the greatest frequency across species (>80%), with the highest concentrations detected in the ileum and rectum of mice and macaques.

### Drug efflux transporter expression and antiretroviral drug distribution

**Fig. 4.**
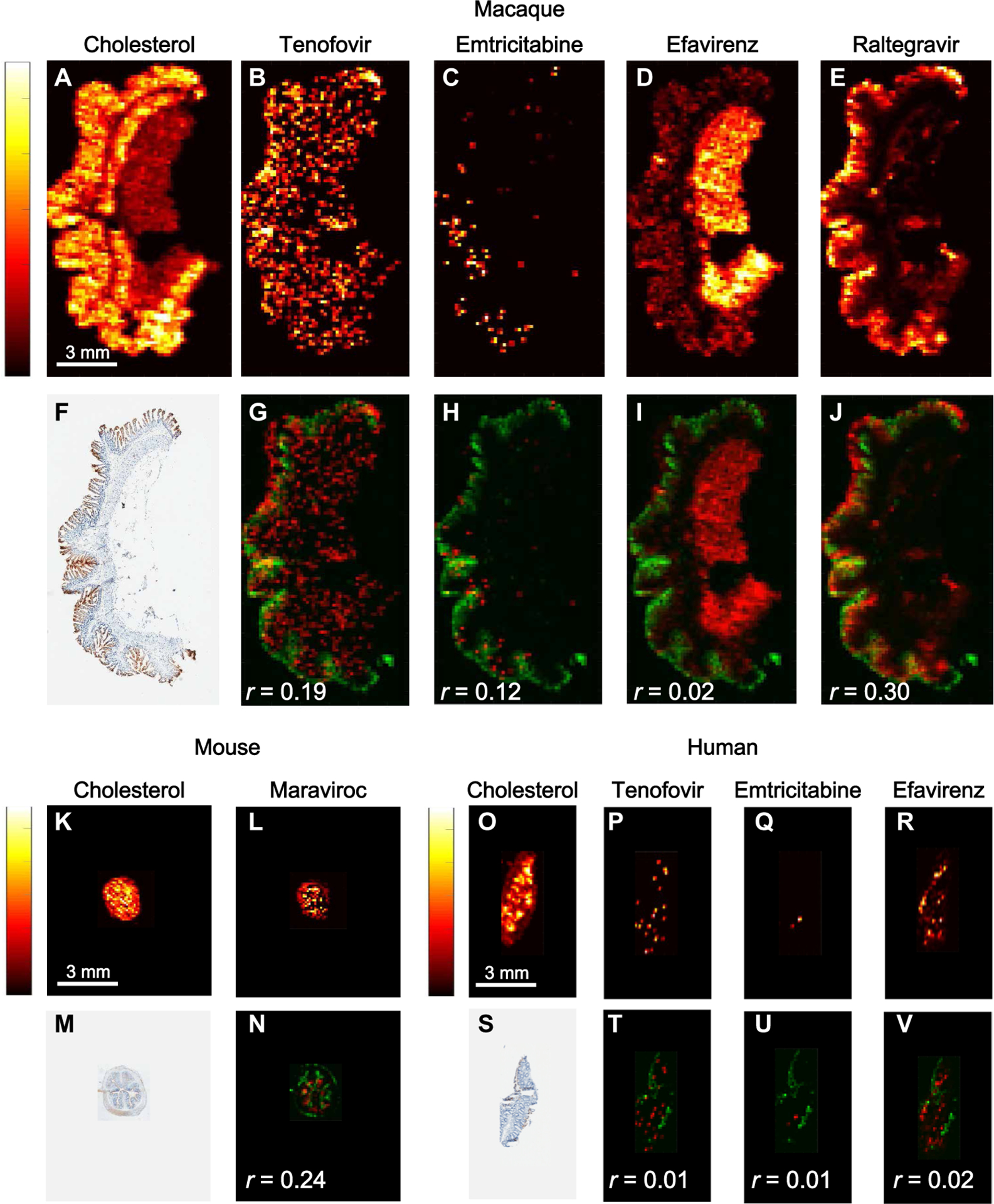
Colocalization of antiretroviral drugs with MDR1 efflux transporter expression in three species. Representative images of antiretroviral drug distribution in a single cross-sectional 10-μm slice of ileum from an uninfected macaque, an HIV-infected humanized BLT mouse, and an HIV-infected human are shown. A total of 24 macaque, 98 mouse, and 10 human tissue slices were imaged by MSI. Antiretroviral drug concentrations in (**B**) to (**E**) are indicated by the color scale on the left: Lighter colors (yellow and white) are associated with higher drug concentrations compared to darker colors (red and black). Endogenous cholesterol distribution is shown in (**A**) to demonstrate tissue orientation for the remaining images. Images (B) to (E) were overlaid with images of MDR1 efflux transporter expression in an adjacent tissue slice (**F**). (**G** to **J**) Overlaid images show drug in red and MDR1 transporter in green. Pearson correlation coefficients (*r*) between these variables are listed in the bottom left corner of each overlaid image. Images of drug distribution alone or overlaid with MDR1 expression are shown for a BLT HIV-infected mouse (J to **M**) and an HIV-infected human (**N** to **U**). Scale bars to the left of each endogenous cholesterol image apply to all antiretroviral drug distribution images from that tissue slice. In the BLT mouse ileum slice shown in (K) and (L), maraviroc was the only drug detected by MSI and is shown overlaid with MDR1 expression (N). In the human ileum tissue section shown in (O) to (R), all three drugs contained in the patient’s antiretroviral regimen were detected by MSI. Images of drug distribution are overlaid with MDR1 expression (S) in (T) to (**V**).

Because drug efflux transporters are known to play a role in the tissue distribution of antiretroviral drugs and have been previously quantified in mouse and macaque tissues (18), we next assessed the potential impact of these efflux transporters by evaluating their distribution compared to the distribution of antiretroviral drugs as quantified by Pearson correlations. Figure 4 shows representative images of the overlap in antiretroviral drug distribution and multi-drug resistance protein 1 (MDR1) efflux transporter expression in mouse, macaque, and human ileum. Figure 4 (B to E) shows heme-corrected MSI images for four different drugs, each showing a distinct distribution pattern in uninfected macaque ileum. When assessed in vitro in a previous study, raltegravir, atazanavir, and maraviroc showed affinity for MDR1 efflux transporter proteins (23). In this study, efavirenz distribution did not appear to be affected by the expression or localization of MDR1 efflux transporters in macaque ileum, because the two variables were not colocalized (*r* = 0.02; Fig. 4I); efavirenz appeared to have crossed the gut mucosa and accumulated in adipose tissue. Conversely, raltegravir accumulated on the luminal surface of the macaque ileum mucosa with limited penetration into the submucosa (Fig. 4E) and colocalized moderately with MDR1 efflux transporters (*r* = 0.3; Fig. 4J). Similarly, tenofovir (Fig. 4B) showed colocalization with MDR1 efflux transporters (Fig. 4G) in macaque ileum despite a lack of evidence supporting tenofovir as a substrate for these proteins.

Representative images of ileum tissue slices from HIV-infected mice and HIV-infected humans are shown in Fig. 4 (K to N and O to V, respectively). MDR1 efflux transporter expression was detected extensively along the luminal surface of mouse gut epithelial cells with low-to-moderate colocalization with antiretroviral drugs (*r* range, −0.1 to 0.5).

## DISCUSSION

Here, we assessed the distribution of antiretroviral drugs in gut tissues across three species and measured the distribution relative to CD3^+^ T cells and expression of HIV or SHIV RNA. Despite dosing with combination drug therapy to a pharmacokinetic steady state, 40 to 60% of CD3^+^ T cells remained unexposed to antiretroviral drugs in gut samples from all three species. The limitations of using conventional methods for small-molecule quantitation in tissue (e.g., LC-MS analysis of whole tissue homogenates) have been acknowledged previously (15, 24), and different models of heterogeneous antiretroviral drug distribution in tissue have been proposed (5). Our data highlight the advantages of using MSI for characterizing tissue pharmacology. We have shown here that antiretroviral drug exposure in some areas where HIV target cells reside may be very small. This is particularly relevant when drawing inferences about target concentrations for antiretroviral drug efficacy, because any proposed drug concentration would need to be achieved in every HIV-infected cell or HIV target cell.

Our use of CD3^+^ T cells to assess antiretroviral drug exposure in the entire T cell population versus a more targeted evaluation of CD4^+^ T cells is a major limitation of our study. Our approach overestimated the number of actual HIV-infected cells or HIV target T cells. Our attempts to identify the smaller CD4^+^ T cell subset were complicated by a high background signal that made discrete cellular identification impossible. However, given that about 50% of the CD3^+^ T cell population did not appear to be exposed to antiretroviral drug and CD4^+^ T cells represent about 50% of the total T cell population in the gut mucosa (25, 26), we predict that some of these unexposed T cells were also CD4^+^ T cells. This assumption is supported by our data showing that viral RNA expression occurred in discrete clusters spread throughout lymphoid aggregates within the ileum and rectum of all three species. Our finding of viral gene expression in the gut during antiretroviral drug therapy is consistent with previous publications (27, 28) and is not surprising given the relatively short duration of drug treatment administered to the mice and macaques in our study. However, our study design was such that we could identify all potential areas of viral replication early during drug treatment in the animals and could determine overlap with drug concentrations. Future work will need to evaluate the relationship between drug exposure and viral gene expression in gut tissues after long-term suppression of plasma HIV RNA by antiretroviral drug therapy. We attempted to address this limitation by examining gut samples from HIV-infected women who had been on combined antiretroviral drug therapy for at least 5 years and who showed suppression of viral loads for more than 1 year. Viral RNA was detected in two of five rectum samples, where 100% of viral RNA was colocalized with at least one drug. We found that antiretroviral drugs, when detectable, were well above reported in vitro IC_90_ values, suggesting that the drug concentrations may have been sufficient to inhibit HIV/SHIV replication. However, because the drug distributions were not homogeneous and T cells moved within tissues, viral replication could still have occurred.

The lack of virus detection in plasma or development of drug resistance during prolonged combined antiretroviral drug therapy in many HIV-infected patients does not support the local tissue viral replication hypothesis (29). It is possible that ongoing viral replication occurs in small pockets of gut tissue and is held in check by high antiretroviral drug concentrations in other tissue regions or in plasma. This is supported by several studies demonstrating the detection of replication-competent HIV DNA in a wide array of tissues collected from HIV-infected patients on combined antiretroviral drug therapy with a suppressed plasma viral load (30, 31). Whereas HIV DNA is a more relevant marker of the latent HIV reservoir, it cannot be ruled out that some proportion of this DNA is undergoing transcription to RNA and eventually is being translated into viral proteins. Given the widespread detection of HIV DNA in tissues, if even a small proportion (<1%) of total HIV DNA led to production of replication-competent virions, then our hypothesis that viral replication occurred in isolated pockets of gut tissue surrounded by efficacious concentrations of antiretroviral drugs would be reasonable.

With our MSI approach, we only captured a static point in time, which did not reflect the dynamic movement of T cells within GALTs. These T cells might be exposed to adequate drug concentrations as they migrate through GALTs, but as they move from areas of high to low drug concentrations, they may end up exposed to much lower intracellular drug concentrations. A future goal of our work is to use MSI to capture intracellular concentrations of antiretroviral drugs in individual T cells.

Although our detection of efavirenz, atazanavir, maraviroc, and raltegravir in gut tissues from three species was robust, we were unable to adequately determine the distribution of tenofovir and emtricitabine, which did not exceed their respective IC_90_ values in most gut samples that we examined. Given that the sensitivity of our MSI method was lower for tenofovir and emtricitabine than the other antiretroviral drugs we tested, these data must be interpreted with caution. In addition, the active intracellular metabolites of these compounds (tenofovir diphosphate and emtricitabine triphosphate) could not be quantified by MSI because preparing tissue slices resulted in their degradation. Therefore, although we quantified these drug metabolites by LC-MS, we did not know the spatial resolution of these drug metabolites in the gut tissue samples. Possibly, these drug metabolites were present in greater abundance and in different distribution patterns than their parent compounds. This may be the case as in a previous study tenofovir diphosphate and emtricitabine triphosphate gut concentrations were high enough after daily oral administration to prevent rectal sexual transmission of HIV (32). Because MSI technology continues to evolve, we anticipate that our detection limits for these drugs will improve, as will our ability to measure stable drug metabolite concentrations in tissues without compromising their distribution.

Our work also shows the potential contribution of drug efflux transporter protein expression in the gut to differences in antiretroviral drug distribution. There were different degrees of colocalization of MDR1 efflux transporter protein expression with the different antiretroviral drugs (Fig. 4). For example, the higher colocalization of MDR1 with raltegravir suggested that the MDR1 transporter may have acted as a barrier to raltegravir distribution into the gut mucosa from the gut lumen (33, 34). The MDR1 efflux transporter appeared to be less important for efavirenz distribution as indicated by the lower colocalization and lower concentration of efavirenz in the gut submucosa. Raltegravir, but not efavirenz, is a known substrate for the MDR1 efflux transporter (35). With the small number of mice and macaques investigated in this study and the large inter-animal variability in MDR1 transporter expression, the colocalization data were often not statistically significant (*P* > 0.05).

Previous work has shown that HIV infection can alter expression of drug transporters by as much as threefold (36) and that HIV infection can alter several pharmacokinetic variables such as intestinal absorption and drug metabolism that may affect antiretroviral drug distribution in tissues (37). Although the antiretroviral drugs examined here are known to use several of the drug efflux transporters that are altered during infection (e.g., raltegravir by MDR1), HIV/SHIV infection did not contribute substantially to drug distribution in ileal or rectal tissues in our study. It is possible that our animals were not infected for long enough for these changes to become apparent (36), but our data do suggest that uninfected animals can be used to assess antiretroviral drug distribution.

Our study has a number of limitations including the technological limitations of MSI and the need for markers of antiretroviral drug efficacy. We generated data from 10-μm tissue slices. The lack of antiretroviral drug detection in some of these tissue slices despite detection with traditional LC-MS methods can be explained by the decreased sensitivity of the IR-MALDESI instrumentation that we used. The relationship between sensitivity and image spatial resolution when using MSI has been noted (24). Compared to LC-MS, the LODs for IR-MALDESI for antiretroviral drugs in tissues are up to 10-fold higher, resulting in decreased sensitivity for detection of some antiretroviral drugs. The inherent decrease in sensitivity with MSI can be explained, in part, by the small tissue area ablated with each laser pulse. Given that each laser pulse ablates a 100-μm area at 10-μm thickness, the tissue volume per analysis is up to 100-fold less than that analyzed by LC-MS using tissue homogenates. In addition, although we demonstrated that tissue architecture could be identified in each tissue slice, our limited sampling of gut tissue may have led to some drug distribution being overlooked. We collected tissues at the end of the drug dosing interval, but tissue penetration of antiretroviral drugs may have been higher or more homogeneous at other pharmacokinetic time points. However, we minimized any sampling confounding by dosing mice and monkeys with drug to plasma steady-state conditions. Total tissue drug concentrations under steady-state conditions are less variable across the dosing interval compared to plasma and so are less likely to differ based on the time of tissue collection (38, 39).

At sampling locations where potential blood contamination was present, we used MSI detection of the blood marker heme b to correct our measurements. This may have resulted in an overcorrection particularly in mouse and human tissues. We attempted to correct this by applying this blood contamination correction to MSI, immunohistochemistry (IHC), and ISH images. The potential contribution from remnant blood to antiretroviral concentrations measured in tissue homogenates further illustrates the limitation of using LC-MS to measure drug concentrations, because there is no capacity to assess proportional antiretroviral drug concentrations in blood by this method.

Although the RNAscope ISH method used here has improved specificity compared to traditional ISH for detecting HIV/SHIV-specific gene expression (40) and the frequency of viral RNA-positive cells may be in good agreement with fluorescence detection of virus-producing cells (41), the aggregate viral RNA detection we obtained may overestimate viral replication by capturing virions trapped on follicular dendritic cells in B cell follicles. Further, the association between cell-associated RNA measured before drug treatment interruption and timing of viral rebound has been demonstrated in other studies (41), but we were not able to discriminate between cell-associated RNA and free virions in our study. By correlating antiretroviral drug distribution to gut regions where active virions could be produced, our results may represent a worst-case scenario. We attempted to overcome this limitation by staining all virus-infected tissues for the viral protein markers p27 (macaque tissue) or p24 (humanized mice and human tissue); however, we were unable to detect these two viral proteins with enough signal to perform a robust analysis with our drug concentration data. Currently, we are investigating measurements of downstream components of the HIV life cycle by ISH and IR-MALDESI MSI.

We compared antiretroviral drug concentrations to their individual IC_90_ values without consideration for the synergistic effects of antiretroviral drugs in combination (Table 3). The antiretroviral drugs evaluated here may have exceeded efficacious concentrations due to the presence of other active drugs at the same gut tissue locations, but we were unable to make those comparisons. This has direct relevance for measuring the proportion of CD3^+^ T cells exposed to at least one antiretroviral drug (Fig. 2 and table S2). Our analysis makes a distinction between CD3^+^ T cells that are not exposed to drug and those that are exposed to at least one drug, but assessments of the whole drug combination regimen could not be performed. Future work will use the MSI signal from endogenous metabolites and lipids to segment and categorize histological features in each tissue (42–45) to allow for cross-experiment comparisons between the whole drug regimens.

The antiretroviral drug concentrations measured in this study represent total rather than unbound drug, whereas drug efficacy is driven by free drug concentrations at the site of action (46). The extent of protein binding and thus free drug concentrations can differ between plasma and tissues for anti-infective agents (46, 47). In some cases, estimates of the free drug in tissues can be accurately extrapolated from plasma drug concentrations (48, 49), but direct measurement of unbound tissue concentrations of drug provides the most useful data for efficacy. Strategies such as ultrafiltration or microdialysis have been used by others to measure unbound drug concentrations in tissues directly (50).

We did find some viral RNA-expressing cells in gut tissue that were not located close to measurable amounts of drug, despite adequate (>IC_90_) exposure elsewhere in the tissue. This does not prove a causal relationship between suboptimal antiretroviral drug exposure and ongoing viral replication given that we likely overestimated both of these variables, but it does suggest the feasibility of applying MSI to elucidate HIV persistence in gut tissue and potentially other tissues.

## MATERIALS AND METHODS

### Study design

The primary objective of this study was to use MSI to characterize antiretroviral drug distribution within gastrointestinal tissues from three animal models (two strains of humanized mice and macaques) and humans. The goal was to quantitatively determine whether antiretroviral drug exposure in gut tissue at a single time point resulted in areas where HIV target CD3^+^ T cells or viral RNA expression did not overlap with drug distribution. Secondary objectives included correlating antiretroviral drug concentrations with drug efflux transporter expression, interspecies comparisons of antiretroviral drug exposure in gut tissues, and assessment of differences in infected and uninfected animals. We hypothesized that antiretroviral drug distribution would be heterogeneous in all three species and could result in tissue regions devoid of any drug. Animals were randomized to an antiretroviral drug treatment regimen, but human subjects were selected on the basis of the regimen they were currently taking. No element of this study was blinded.

### Animal models

We used three animal models from two species: the hu-HSC-Rag (*n* = 36) and BLT (*n* = 13) humanized mouse models and a rhesus macaque NHP model (*n* = 12). Detailed descriptions of the animals used for this study, including infection and dosing information, as well as plasma concentrations, have been reported elsewhere (18). Briefly, female humanized mice from both models were dosed orally for 6 to 10 days with one of several antiretroviral regimens (table S4). In addition, male rhesus macaques (*Macaca mulatta*) were dosed for 10 days with tenofovir and emtricitabine (both subcutaneously) and orally with either maraviroc + atazanavir or efavirenz + raltegravir. Antiretroviral doses, routes of administration, and duration of therapy were chosen to mimic commonly used treatment doses in these models and achieve pharmacokinetic steady-state conditions for all agents.

About one-half the animals in each dosing cohort were infected with HIV (humanized mice) or SHIV (macaques) to assess the relationship between antiretroviral tissue disposition and potential ongoing HIV infection using HIV_Bal D7_ (hu-HSC-Rag mice), HIV_JRcsf_ (BLT mice), or RT-SHIV_mac239_ (macaques) as previously described in detail (18). Animals were euthanized and underwent necropsy about 24 hours after the final antiretroviral dose. Plasma was collected, and tissues were removed from the body, cut into two about equalsized pieces, and snap-frozen on dry ice. All animal experiments were performed in accordance with locally approved institutional animal care and use committee (IACUC) protocols.

### Clinical trial design and subject demographics

This study enrolled five HIV-positive women between 18 and 65 years of age (inclusive) with intact gastrointestinal and genital tracts, who had undetectable plasma HIV RNA (<50 copies/ml) within 3 months preceding the study. Participants were recruited from the Infectious Diseases Clinic at University of North Carolina (UNC) Chapel Hill on the basis of receiving one of the study regimens (tenofovir disoproxil fumarate/emtricitabine as a fixed dose combination plus either efavirenz, raltegravir, maraviroc, or atazanavir) as part of their ongoing clinical care.

Subjects could not have a history of gastrointestinal disease (e.g., Crohn’s disease, irritable bowel syndrome, ulcerative colitis, diverticulitis, and colon cancer) or have a history of gastrointestinal surgery. All subjects had a negative serum pregnancy test at screening and negative urine pregnancy tests on days of sampling and used at least one of the following methods of contraception from the screening visit through 72 hours before inpatient admission (at which time the women were asked to remain abstinent until after their follow-up visit): systemic hormonal contraceptive (oral, depot, transdermal, or implant), intrauterine device (IUD) placed at least 1 month before study enrollment, bilateral tubal ligation (sterilization), vasectomized male partners, or condom with spermicide.

Additional inclusion criteria included BMI of about 18 to 37 kg/m^2^, a total body weight of >45 kg (99 lbs), and evidence of a personally signed and dated informed consent document indicating that the subject has been informed of all pertinent aspects of the trial and was willing and able to comply with scheduled visits, treatment plan, laboratory tests, and other trial procedures. Further, women could not be receiving any known CYP3A4 inducers (rifampin, carbamazepine, and St. John’s wort) or inhibitors [ketoconazole, non–dihydropyridine (DHP) calcium channel blockers, and macrolide antibiotics] other than those contained in their HIV regimen for at least 6 months before enrollment. Subjects had to be willing to abstain from sexual intercourse, douching, and all intrarectal objects and products for at least 72 hours before enrollment until study completion; have a negative hepatitis B surface antigen test as documented on screening laboratories; could not be actively involved in the conception process; were able to swallow pills; and have no allergies to any component of the study products (i.e., bowel preparation regimen).

Participants were excluded if they had any clinically relevant abnormal screening evaluations or relevant comorbidities, untreated sexually transmitted infections (rectal chlamydia or gonorrhea, syphilis, and trichomonas), were pregnant or lactating, or tested positive for any drugs of abuse that would increase study risks. Additional exclusion criteria included receiving CYP3A4 inducers or inhibitors (other than those contained in their HIV regimens) in the previous 6 months, receiving any investigational drug in the last 4 months, history of inflammatory bowel conditions (Crohn’s disease), or not using an approved method of contraception (systemic hormonal contraception, IUD, bilateral tubal ligation, vasectomized male partner, condom plus spermicide, female-only sex partners, or 3 months of abstinence before enrollment).

Further, subjects were excluded if they had a history of febrile illness within 5 days before enrollment, active hepatitis B infection as determined by positive hepatitis B surface antigen (HBsAg), any laboratory chemistry or hematology result grade 3 or greater according to the Division of AIDS (DAIDS) Laboratory Grading Tables, history of regular alcohol consumption exceeding 14 drinks [1 drink = 5 ounces (150 ml) of wine or 12 ounces (360 ml) of beer or 1.5 ounces (45 ml) of spirits] per week, participation in a clinical trial involving rectal biopsies within 6 months preceding enrollment, blood donation of about 1 pint (500 ml) within 56 days before dosing, history of sensitivity to heparin or heparin-induced thrombocytopenia, or allergy to latex or was unwilling or unable to comply with the dietary and concomitant drug restrictions in regard to study drug administration as outlined in the study procedures and prohibited medication sections. All study activities were approved by the UNC Biomedical Institutional Review Board, and all activities were conducted in accordance with all International Council for Harmonisation of Technical Requirements for Pharmaceuticals for Human Use (ICH) and Good Clinical Practice (GCP) industry standards. All participants provided written informed consent before study procedures were performed.

After participant education, informed consent, and screening for study eligibility, participants were assigned to the treatment arm associated with the HIV regimen they were receiving. Within 42 days of screening, participants were admitted to the NC Translational and Clinical Sciences Institute (NC TraCS) Clinical and Translational Research Center (CTRC) for a 36-hour inpatient sampling visit. For the 7 days before admission, participants were asked to follow a strict low-fiber diet in preparation for a colonoscopy. Once admitted, participants were given only clear liquids until becoming NPO (nothing by mouth) 4 hours before the scheduled colonoscopy. Bowel preparation included an oral laxative (two 5-mg bisacodyl tablets) followed by 227.1 g of polyethylene glycol 3350 in a 1-gallon solution (GoLYTELY). The preparation was divided into two separate 2-hour blocks: the first beginning 18 hours before the scheduled procedure and the second half beginning 6 hours before the procedure. Before the procedure, a single 3-ml paired blood sample was taken and processed for plasma. During the procedure, 10 biopsies each were taken from the terminal ileum and rectum. Subjects were monitored for 6 hours after the procedure, before discharge, with follow-up visits within 14 days of discharge. An analysis of previous studies from our group in subjects receiving raltegravir or darunavir who underwent similar procedures showed an about 30% decrease in both gut tissue and plasma concentrations (as measured by traditional LC-MS) versus subjects who had not received a complete bowel preparation.

Safety assessments were conducted on each day of the inpatient visit and during follow-up. Adverse events were evaluated using a standard questionnaire, with grading according to the DAIDS adverse events grading table version 2.0. Women of childbearing potential were screened for pregnancy at each visit.

### Human tissue collection

Ileal and rectal biopsies were collected during colonoscopy with Radial Jaw 4 large capacity biopsy forceps (Boston Scientific, Boston, MA). All tissues were immediately snap-frozen on dry ice, then placed in aluminum foil pouches, and stored at −80°C until analysis. Whole blood was collected in 3-ml EDTA tubes and centrifuged at 3000 rpm for 10 min at 4°C. Plasma was aliquoted into a 2-ml cryovial and stored at −80°C until analysis.

### Tissue slices

To generate serial sections for multimodal analysis, tissues were sliced frozen at 10-μm thickness using a cryostat (Leica Biosystems, Wetzlar, Germany) and thaw-mounted onto glass microscope slides in the following order: 8 slices for IHC, 2 slices for MSI (1 for analysis and 1 backup), 2 slices for LC-MS, and 15 to 20 slices for ISH. Macaque tissues were mounted on optimal cutting temperature (OCT) compound and sliced individually; however, the small size of the mouse tissues and human biopsies (2- to 5-mm cross sections) precluded this method. Instead, tissues were grouped by dosing cohort and mounted within a 2:1 gelatin carboxymethylcellulose gel block, which was snap-frozen and stored at −80°C. Dosing groups for mice each contained *n* = 6, and six tissues were included in a single gelatin block (e.g., six ileums from the atazanavir-only dosing group in a single block and six rectums from the atazanavir-only dosing group in a single gelatin block). For the human samples, ileum and rectum from all five women were included in single blocks (one block for ileum and one block for rectum). Each frozen gel block was mounted on OCT, sliced, and thaw-mounted, allowing for mounting and analysis of up to six mouse tissues simultaneously (51). Analysis of embedded and non-embedded tissues suggests that the gel embedding process does not alter antiretroviral distribution within tissues (fig. S1).

### MSI and quantification

The glass microscope slide containing the thaw-mounted tissue was placed into the IR-MALDESI MSI source chamber and maintained at −10°C. Relative humidity inside the chamber was reduced to <6% to allow for sample cooling without condensation of water vapor, and then humidity was increased to deposit a layer of ice across the entire stage. Tissues were ablated with two pulses of a mid-IR laser (IR-Opolette 2371, Opotek, Carlsbad, CA) with a 100-μm spot-to-spot distance. Ablated molecules were ionized by orthogonal electrospray using 0.2% formic acid in 50:50 methanol water as an electrospray solvent and sampled into a Thermo Fisher Scientific Q Exactive mass spectrometer (Bremen, Germany) for analysis in positive ion mode. Because the detection of certain analytes (e.g., tenofovir and efavirenz) was found to be increased in humans by probing for negative ions, polarity switching was used during these experiments using 5 mM ammonium acetate in 50:50 methanol water as the electrospray solvent. Raw data from each voxel were converted to the mzML format with MSConvert (ProteoWizard) and then to the imzML format for interrogation using MSiReader, which allows for generation of images of antiretroviral distribution across the tissue slice (52, 53).

Absolute quantitation of antiretroviral concentration was achieved by spotting a series of calibration standards (of known antiretroviral concentration) onto a nondosed “blank” tissue slice from identical tissue matrices from each species (ileum or rectum; BioreclamationIVT, Baltimore, MD). Each calibration standard (100 nl) was spotted onto the tissue, allowed to air dry, then placed inside the source chamber, and analyzed in an identical manner to the samples. A new calibration tissue was analyzed every day that sample analysis occurred to account for interday variability in run conditions (electrospray stability, relative humidity, thickness of ice layer, etc.). Calibration tissues were analyzed in MSiReader, where the summed voxel intensity over each calibration spot was plotted against the known antiretroviral concentration to generate a calibration curve. The slope and intercept of this curve was applied to the summed voxel intensity value for each antiretroviral over the entire area of the corresponding sample to generate an absolute concentration (15). To quantify antiretroviral concentration in mouse tissues, a single calibration spot was applied to a blank mouse tissue and analyzed in tandem with macaque calibration tissues. The voxel intensity value for the mouse calibration spot was used to adjust the slope and intercept of the NHP calibration curve (to account for response differences between tissues from different species), and the adjusted calibration curve was applied to mouse samples. Resulting ng/slice concentrations were converted to μg/g using the known area of each tissue slice measured in MSiReader, depth of each tissue (10 μm), and an assumed tissue density of 1.06 g/ml.

For the purposes of inclusion in this manuscript, “representative” tissue sections were selected primarily on the basis of morphology, slice quality, and quality of IR-MALDESI image. For example, when possible, we include tissue slices that show a complete cross section from gut lumen through the muscularis, with minimal tissue folding or tearing in the IHC or ISH slice. However, our results are based on analysis of all slices analyzed regardless of aesthetics, and we report descriptive statistics (median and range) for all variables. To assess interslice variability, we a priori took multiple consecutive slices from each tissue for IR-MALDESI analysis, with the idea that several backup slices would be available in the event of experimental error. Several of these backup slices were analyzed and compared to the original data, with good qualitative correlation in distribution.

### LC-MS analysis and comparison to MSI

Plasma and tissue antiretroviral quantification of each sample was performed using LC-MS methods as described previously (19). Plasma samples, calibration standards, and quality control samples underwent protein precipitation followed by LC-MS/MS. Internal standard was added to plasma and mixed with 600 μl of acetonitrile. Samples were vortexed and centrifuged, and then the supernatant was diluted with 50:50 methanol/water. For tissues, 1 ml of ice-cold 70:30 acetonitrile/water was added to sample tubes containing a serial 10-μm section from each sample. Samples were sonicated for 10 min with calibration standards and quality control samples. Separation for both matrices occurred on a Shimadzu high-performance liquid chromatography system and an AB SCIEX API 5000 mass spectrometer (AB SCIEX, Foster City, CA, USA) equipped with a turbo spray interface. The dynamic range of the plasma assay ranged from 1 to 20,000 ng/ml for tenofovir, atazanavir, and efavirenz; 1 to 8000 ng/ml for maraviroc; and 8 to 20,000 ng/ml for emtricitabine and raltegravir. The dynamic range for tissues is from 0.1 to 50 ng/ml. The precision and accuracy of the calibration standards and QC samples were within the acceptable range of 15%. Tissue concentrations were reported as ng/slice and converted to μg/g using an assumed tissue density of 1.06 g/ml.

### Immunofluorescence and IHC

Drug efflux transporter localization was identified using IHC as described previously (18). Dual immunofluorescence (IF) on frozen humanized mouse and NHP sections was performed on the Bond fully automated slide staining system (Leica Microsystems) using Bond Polymer Refine Detection kit (DS9800). Slides were allowed to sit at room temperature for 30 min and then fixed in 10% formalin for 15 min. They were then placed in Bond wash solution (AR9590). Antigen retrieval was done at 100°C in Bond epitope retrieval solution 2 (pH 9.0; AR9640) for 10 min. Staining was performed first using CD4 1F6 antibody (clone BC/1F6, Abcam) at 1:50 dilution for 1 hour with Bond polymer and post-primary reagents and Cy5 fluorochrome (PerkinElmer) for 15 min. Antigen retrieval was done at again 100°C in Bond epitope retrieval solution 2 (pH 9.0; AR9640) between protocols. Slides were then stained with CD3 (clone LN10, Leica) Ready-to-Use antibody for 15 min and Dako Envision mouse secondary for 30 min. Cy3 fluorochrome (PerkinElmer) was applied for 15 min. IF slides were counterstained with Hoechst 33258 (Invitrogen, Carlsbad, CA) and mounted with ProLong Gold antifade reagent (P36934, Life Technologies).

### In situ hybridization

Fifteen to 20 serial sections from each tissue were evaluated for HIV/SHIV RNA expression using RNAscope ISH (54). Before beginning the RNAscope procedure, slides were fixed in 4% paraformaldehyde at 4°C for 15 min followed by dehydration with graded ethanol washes (50, 70, and 100% ethanol for 5 min each). Detailed methodology for the RNAscope procedure is described elsewhere (54). Briefly, slides were boiled to retrieve epitopes in P2 buffer (Advanced Cell Diagnostics, ACD) for 30 min followed by peroxidase blocking for 10 min at room temperature, rinsing with double-distilled water, dehydrating with 100% ethanol for 5 min, and then air-drying. Slides were then incubated for 10 to 20 min at 40°C with protease digestion solution from ACD (P3). After protease digestions, slides were rinsed with double-distilled water and incubated with HIV clade B or SIV_mac239_ ACD probes for 2 hours at 40°C. Slides were then washed in 0.5× ACD wash buffer and incubated in amplification reagents according to the RNAscope 2.5 HD detection protocol (54). All reagents used in the hybridization process were obtained from Advanced Cell Diagnostics (Newark, CA) and used according to the manufacturer’s protocol with some minor adaptations (54). After counterstaining slides with hematoxylin, slides were mounted in clear mount and coverslipped. Staining was performed on all 15 to 20 slides to ensure detection of positive signal. Results were compared across slides, and little variability was found. Thus, colocalization was performed using the tissue slice most adjacent to the slice used for MSI.

### Image colocalization

A schematic of the colocalization workflow is shown in fig. S3. For a given sample (fig. S3A), MSiReader was used to export voxel intensity matrices for cholesterol, heme, and each antiretroviral of interest across the entire tissue slice into MATLAB; IF, ISH, and IHC samples were scanned as described above and downsampled to match the resolution of the MSI data (fig. S2). Off-tissue response was eliminated by using cholesterol signal to mask antiretroviral response such that only on-tissue signal was shown. To eliminate the confounding effect of antiretrovirals contained within the vasculature, antiretroviral responses were again masked on the basis of heme distribution (fig. S3B) to show only the antiretroviral signal that localized outside the microvasculature. To ensure that MSI-derived images and IF/IHC/ISH images were appropriately aligned before colocalization, co-registration was performed by rigid transformation of the moving cholesterol image using the background 4′,6-diamidino-2-phenylindole (DAPI) stain as a fixed reference (fig. S3C). The resulting transform matrix was then applied to all antiretroviral images so that every antiretroviral image was identically oriented. Last, the heme-corrected transformed antiretroviral images were overlayed with the variable of interest (CD3, MDR1, etc.) to generate a fused image (fig. S3D) containing both the antiretroviral (in red) and the variable of interest (in green).

To evaluate concentrations proximate to relevant variables, antiretroviral intensity from a given voxel was multiplied by the total slice concentration determined from the quantitative experiments and corrected on the basis of the proportional contribution of that voxel to the total antiretroviral signal detected in the slice. Descriptive statistics (median and range) were generated on the basis of these per-voxel antiretroviral concentrations for areas of colocalization with the variable of interest (CD3, RNA, etc.). Per-voxel concentrations were then thresholded on the basis of the maximum in vitro IC_90_ value reported in the package insert for each drug to generate binary images, showing antiretroviral concentration above and below the IC_90_ value. Where ranges of IC_90_ values could be found, we used the highest value to provide conservatives estimates of adequate antiretroviral exposure.

### Statistical analysis

Descriptive statistics (median, range) of plasma and tissue drug concentrations as well as correlation coefficients were generated for each drug in humanized mice and NHPs. Pearson correlation was used to assess potential effects of drug efflux transporter expression on antiretroviral drug distribution. Sample sizes were chosen to provide *n* = 6 for each animal dosing group, providing statistical power to detect 15% differences in antiretroviral drug penetration between groups. Because of evolving drug treatment regimens among our clinic population, we did not meet our recruitment goal of *n* = 6 for each treatment group in the clinical study and thus did not meet our prespecified power requirement. Comparisons between animal models and between anatomical compartments were performed using Kruskal-Wallis analysis of variance (ANOVA) on ranks. *P* < 0.05 was considered statistically significant.

## Supplementary Material

Supplemetary material Fig. S1. Effect of gelatin embedding on antiretroviral drug distribution.
Fig. S2. Resolution matching of microscopy and MSI data.
Fig. S3. Image colocalization workflow.
Table S1. Subject demographics.
Table S2. Human plasma and tissue antiretroviral drug concentrations.
Table S3. Proportion of CD3^+^ T cells exposed to at least one antiretroviral drug.
Table S4. Dosing of animals.
Data file S1. Individual-level data for tables.

## Figures and Tables

**Table 1.** Percent CD3^+^ T cells not colocalizing with any antiretroviral drug.

	BLT (*n* = 13)	hu-HSC-Rag (*n* = 36)	Macaque (*n* = 12)	Human (*n* = 5)	*P*
Ileum	50 (10–100)	100 (20–100)	40 (10–90)	40 (30–50)	<0.01
Rectum	40 (10–100)	100 (20–100)	50 (20–80)	40 (20–70)	<0.01

Data are median and range.

**Table 2.** Percent viral RNA expression colocalizing with antiretroviral drug.

	BLT (*n* = 7)	hu-HSC-Rag (*n* = 18)	Macaque (*n* = 6)	Human (*n* = 5)*	*P*
Ileum	10 (0–100)	0 (0–100)	80 (40–90)	90 (80–90)	<0.01
Rectum	50 (20–100)	0 (0–100)	60 (20–80)	100 (100–100)	<0.01

Data are median and range.

* Viral RNA was detected in rectal samples from only two of five women.

**Table 3.** Drug concentrations proximate to viral RNA.

Drug	Ileum				Rectum				Reported in vitro IC_90_
	Mice		All macaques	All humans	Mice		All macaques	All humans	
	BLT	hu-HSC-Rag			BLT	hu-HSC-Rag			
TFV	49 (0–1,782)	0 (0–93)	97 (0.4–151)	0 (0–340)	844 (0–1,499)	0 (0–5,152)	80 (0–182)	0 (0–158)	2,980
TFVdp*	1.7 (0–4.1)	0.8 (0–7)	0.4 (0.03–2.8)	21 (11–65)	0.003 (0–0.1)	0.07 (0–11)	0.2 (0.03–17)	17 (9.4–23)	1.7
FTC	0 (0–96)	ND	70 (1–206)	ND	0 (0–37)	0 (0–41)	0 (0–66)	0 (0–6)	84
FTCtp*	0.09 (0–0.2)	ND	0.02 (0.005–0.05)	0.7 (0.1–6.5)	ND	ND	0.001 (0–0.2)	3.3 (1.7–7.7)	2.8
RAL	ND	ND	12,887 (4,420–21,353)	18,632	ND	ND	6,891 (2,877–10,906)	387	10
EFV		ND	358 (215–501)	24,687		0 (0–21)	385 (268–501)	15,178	10
MVC	5,739 (0–14,872)	0 (0–4,776)	3,199 (1,059–5,822)	1	3,117 (1,589–8,759)	0 (0–724)	3,990 (957–4,625)	ND	6
ATZ	2,972 (0–28,616)	0 (0–117,819)	1,987 (1,152–3,674)	ND	596 (0–2,794)	0 (0–23,632)	1,068 (980–8,861)	1,832	11

Data are median and range. Conversion from ng/ml to ng/g was performed using an assumed tissue density of 1.06 g/ml. IC_90_ values for each drug are reported from the following references: tenofovir (55), tenofovir diphosphate (56), emtricitabine (57), emtricitabine triphosphate (58), efavirenz (59), maraviroc (60), atazanavir (61), raltegravir (62). ND, analyte not detected in any tissue; TFV, tenofovir; TFVdp, tenofovir diphosphate; FTC, emtricitabine; FTCtp, emtricitabine triphosphate; RAL, raltegravir; EFV, efavirenz; MVC, maraviroc; ATZ, atazanavir.

* Tenofovir diphosphate and emtricitabine triphosphate concentrations (detected by LC-MS) and IC_90_ values are reported as ×10^5^ fmol/g of tissue.
