# Supplementary material for: Heterogeneous antiretroviral drug distribution and HIV/SHIV detection in the gut of three species
Source: Sci Transl Med. Author manuscript; Available in PMC 2021 Jul 12. (PMC8273920; doi:10.1126/scitranslmed.aap8758)
Supplement: Supplemetary material — Fig. S1. Effect of gelatin embedding on antiretroviral drug distribution. Fig. S2. Resolution matching of microscopy and MSI data. Fig. S3. Image colocalization workflow. Table S1. Subject demographics. Table S2. Human plasma and tissue antiretroviral drug concentrations. Table S3. Proportion of CD3+ T cells exposed to at least one antiretroviral drug. Table S4. Dosing of animals. Data file S1. Individual-level data for tables. [file NIHMS1718903-supplement-Supplemetary_material.pdf]

Science Translational Medicine
AAAS

stm.sciencemag.org/cgi/content/full/11/499/eaap8758/DC1

Supplementary Materials for

Heterogeneous antiretroviral drug distribution and HIV/SHIV detection in the gut of three species

Corbin G. Thompson, Elias P. Rosen, Heather M. A. Prince, Nicole White, Craig Sykes, Gabriela de la Cruz, Michelle Mathews, Claire Deleage, Jacob D. Estes, Paige Charlins, Leila R. Mulder, Martina Kovarova, Lourdes Adamson, Shifali Arora, Evan S. Dellon, Anne F. Peery, Nicholas J. Shaheen, Cynthia Gay, David C. Muddiman, Ramesh Akkina, J. Victor Garcia, Paul Luciw, Angela D. M. Kashuba*

*Corresponding author. Email: akashuba@unc.edu

Published 3 July 2019, *Sci. Transl. Med.* **11**, eaap8758 (2019)
DOI: 10.1126/scitranslmed.aap8758

The PDF file includes:

Fig. S1. Effect of gelatin embedding on antiretroviral drug distribution.
Fig. S2. Resolution matching of microscopy and MSI data.
Fig. S3. Image colocalization workflow.
Table S1. Subject demographics.
Table S2. Human plasma and tissue antiretroviral drug concentrations.
Table S3. Proportion of $CD3^+$ T cells exposed to at least one antiretroviral drug.
Table S4. Dosing of animals.

Other Supplementary Material for this manuscript includes the following:

(available at stm.sciencemag.org/cgi/content/full/11/499/eaap8758/DC1)

Data file S1 (Microsoft Excel fromat). Individual-level data for tables.

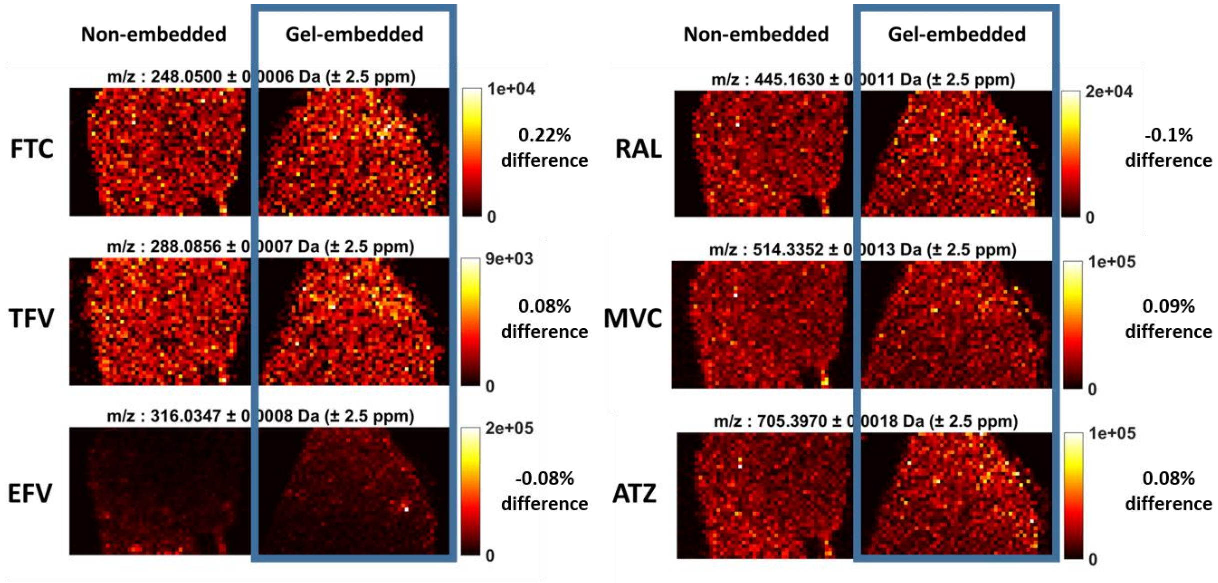

Fig. S1. Effect of gelatin embedding on antiretroviral drug distribution. Raw MSI images are shown for the six ARVs evaluated in this study from ARV-incubated tissue that was divided in two and analyzed with and without gelatin embedding. Signal abundance for each ARV is shown in the scale bar to the right of each image.

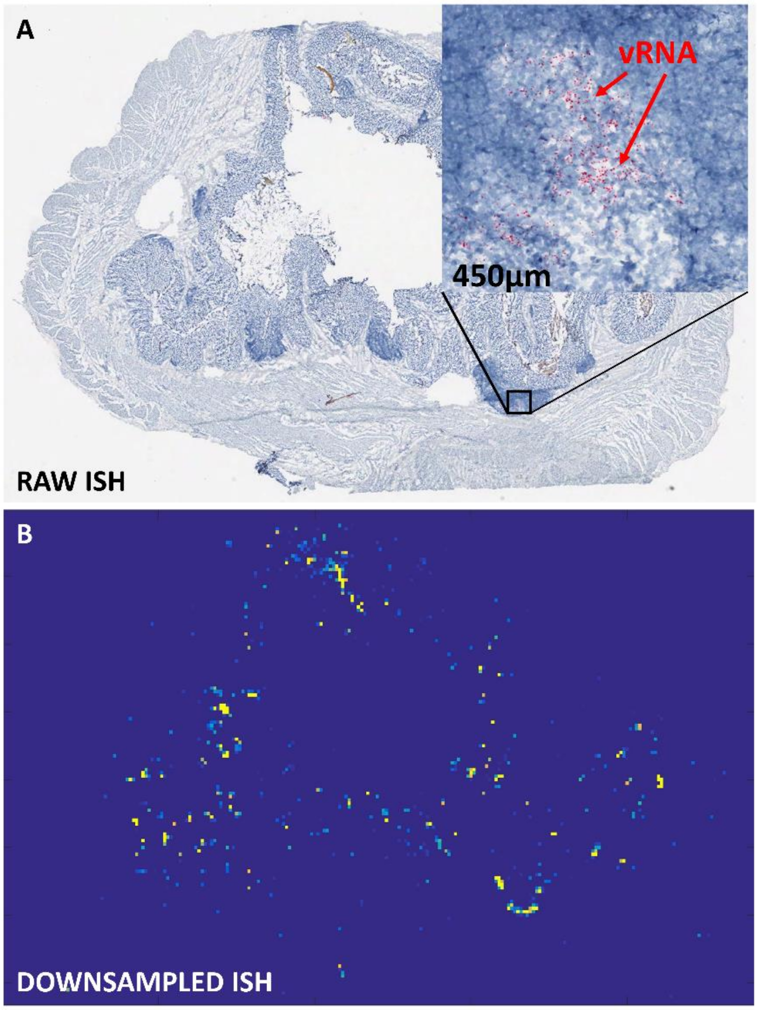

Fig. S2. Resolution matching of microscopy and MSI data. Raw in situ hybridization (ISH) image of a non-human primate rectum (A). Positive vRNA staining is shown in red in the insert of A. Raw ISH images were thresholded based on positive staining and down-sampled to match the resolution of MSI data (B), with brighter colors (yellow) representing positive signal.

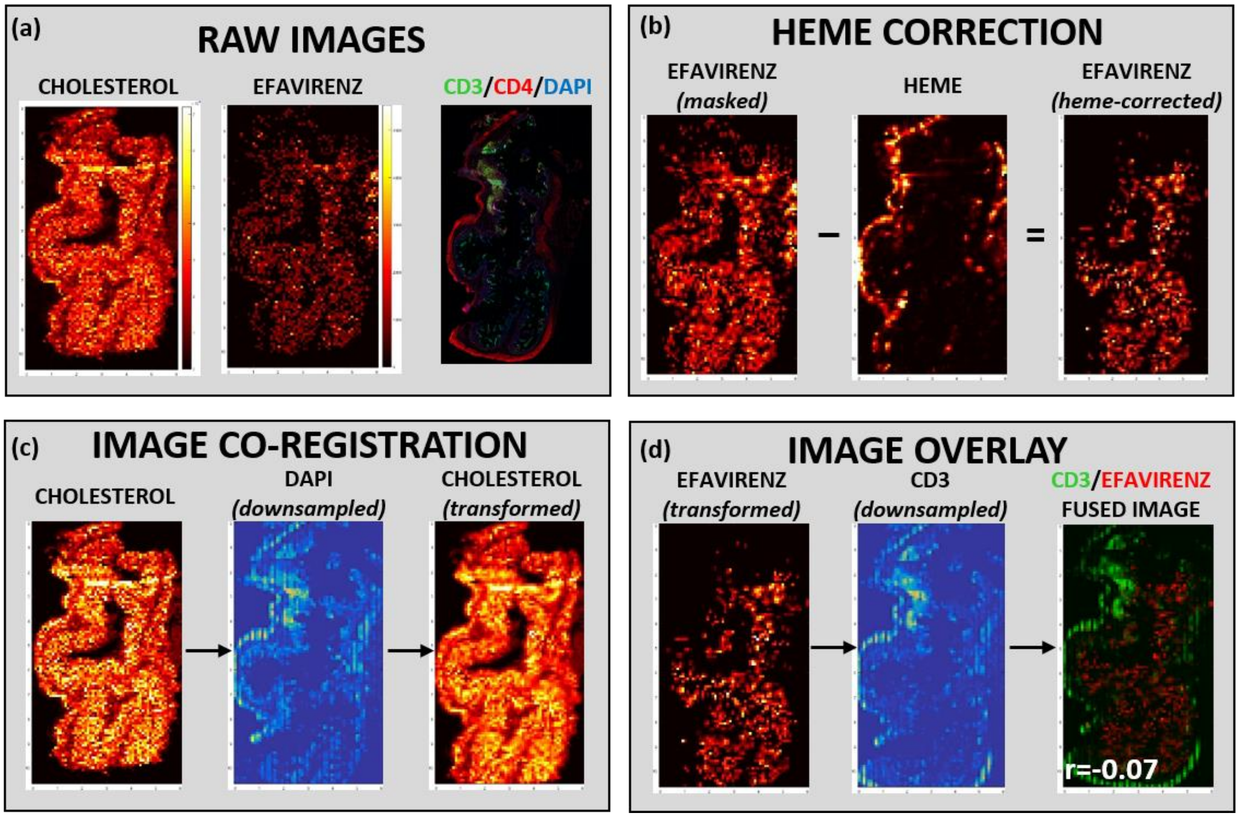

Fig. S3. Image colocalization workflow. (a) Representative raw IR-MALDESI (cholesterol and efavirenz) and IF (CD3, CD4, DAPI) images. (b) Total ARV signal is masked based on corresponding heme distribution. (c) Co-registration of cholesterol and DAPI image leading to transformed cholesterol. (d) Overlay of ARV and variable of interest (CD3 in figure) to form fused image of ARV (red) and CD3 (green), with correlation coefficient shown.

Table S1. Subject demographics.

	EFV (n=1)	RAL (n=1)	ATZ (n=2)	MVC (n=1)
**Age (years)**	55	49	50 (47, 53)	56
**Race**				
**Caucasian**			1	
**African-American**	1	1	1	1
**Menopause Status**				
**Pre**	1		1	
**Post**		1	1	1
**BMI (kg/m2)**	39.1	44.8	37.7 (35.6, 39.8)	26.7
**CD4 (cells/mm3)**	782	753	862 (651, 1074)	672
**Plasma HIV RNA**	ND	ND	ND	ND
**Time on Current Regimen (years)**	7	5	9.5 (8, 11)	8
**Time Since Diagnosis (years)**	26	6	20.5 (17, 24)	18

*data shown are median and range

ND=not detected

Table S2. Human plasma and tissue antiretroviral drug concentrations.

	Emtricitabine n=5		Tenofovir n=5		Efavirenz n=1		Raltegravir n=1		Maraviroc n=1		Atazanavir n=2	
Plasma (ng/mL)	114 (63, 612)		77 (36, 236)		1140		368		89		1552 (413, 2690)	
	Ileum	Rectum	Ileum	Rectum	Ileum	Rectum	Ileum	Rectum	Ileum	Rectum	Ileum	Rectum
Tissue (ng/g)	1019 (389, 2762)	600 (146, 1306)	9802 (2655, 17281)	1736 (1385, 3145)	2476	3044	65645	84072	6246	2614	18860	15107 (13284, 16929)
Tissue:Plasma Ratio	7 (2, 12)	3 (1, 9)	87 (35,297)	41 (6,52)	5	6	189	242	74	31	30 (6,55)	24 (5,43)

*Data shown are median and range

Table S3. Proportion of $CD3^+$ T cells exposed to at least one antiretroviral drug.

Drug	Ileum				Rectum			
	Mice		All Macaques	All Humans	Mice		All Macaques	All Humans
	BLT	hu-HSC-Rag			BLT	hu-HSC-Rag		
**TFV**	0 (0-0.4)	ND	0.1 (0-0.5)	0.2 (0-0.5)	0.3 (0-0.7)	0.5 (0-0.8)	0.1 (0-0.4)	0.1 (0-0.2)
**FTC**	0 (0-0.1)	ND	0 (0-0.4)	0 (0-0.7)	ND	ND	0.1 (0-0.2)	0.1 (0-0.8)
**RAL**	0 (0-0.8)	ND	0.4 (0.2-0.8)	0.5	0 (0-0.8)	0 (0-0.6)	0.4 (0.2-0.7)	0.6
**EFV**		ND	0.6 (0-0.9)	0.6		0 (0-0.3)	0.5 (0.3-0.8)	0.3
**MVC**	0.1 (0-0.8)	0 (0-0.7)	0.5 (0.2-0.6)	0.6	0.3 (0-0.8)	0 (0-0.8)	0.3 (0.2-0.6)	0.3
**ATZ**	0.1 (0-0.8)	0 (0-0.8)	0.4 (0.2-0.5)	0.6	0.2 (0-0.7)	0 (0-0.7)	0.2 (0-0.4)	0.6

*data shown are median and range
ND=not enough detectable samples to make comparison

TFV= tenofovir, FTC=emtricitabine, RAL=raltegravir, EFV=efavirenz, MVC=maraviroc, ATZ=atazanavir

Table S4. Dosing of animals.

Dosing Regimen	MICE				MACAQUES			
	BLT		hu-HSC-Rag		TFV/FTC/ATZ/MVC		TFV/FTC/EFV/RAL	
	+	-	+	-	+	-	+	-
**EFV**			N=6	N=6				
**ATZ**			N=6	N=6				
**TFV/FTC/RAL/MVC**			N=6	N=6				
**TFV/FTC/RAL/MVC/ATZ**	N=7	N=6						
**CONTROL**	N=2	N=2	N=3	N=2				
**TFV/FTC/ATZ/MVC**					N=3	N=3	N=3	N=3
**TFV/FTC/EFV/RAL**					N=3	N=3	N=3	N=3

TFV= tenofovir, FTC=emtricitabine, RAL=raltegravir, EFV=efavirenz, MVC=maraviroc, ATZ=atazanavir
